# Vector vortex beams sorting of 120 modes in visible spectrum

**DOI:** 10.1515/nanoph-2023-0482

**Published:** 2023-10-04

**Authors:** Qi Jia, Yanxia Zhang, Bojian Shi, Hang Li, Xiaoxin Li, Rui Feng, Fangkui Sun, Yongyin Cao, Jian Wang, Cheng-Wei Qiu, Weiqiang Ding

**Affiliations:** Institute of Advanced Photonics, School of Physics, Harbin Institute of Technology, Harbin 150001, China; School of Physics, Harbin Institute of Technology, Harbin 150001, China; Department of Electrical and Computer Engineering, National University of Singapore, Singapore 117583, Singapore; Collaborative Innovation Center of Extreme Optics, Shanxi University, Taiyuan 030006, Shanxi, China

**Keywords:** vector vortex beam, mode demultiplexing, optical neural network

## Abstract

Polarization (*P*), angular index (*l*), and radius index (*p*) are three independent degrees of freedom (DoFs) of vector vortex beams, which have found extensive applications in various domains. While efficient sorting of a single DoF has been achieved successfully, simultaneous sorting of all these DoFs in a compact and efficient manner remains a challenge. In this study, we propose a beam sorter that simultaneously handles all the three DoFs using a diffractive deep neural network (D^2^NN), and demonstrate the robust sorting of 120 Laguerre–Gaussian (LG) modes experimentally in the visible spectrum. Our proposed beam sorter underscores the considerable potential of D^2^NN in optical field manipulation and promises to enhance the diverse applications of vector vortex beams.

## 1 Introduction

Since 1992, Allen et al. discovered that a light beam with the helical phase structure of exp(i*lφ*) carries a determined orbital angular momentum (OAM) of *l*ℏ per photon (*l*, the topological charge, usually is an integer) [1]. Superior to the spin angular momentum *σ*ℏ (with *σ* = ±1), any spatial basis has an infinite number of dimensions [2]. OAM has an infinite number of dimensions in principle, as denoted by *l*. OAM beams have been widely adopted in diverse applications with unprecedented performances due to the merit of the helical phase structure, including advanced optical trapping [3–6], resolution enhanced microscopy [7–9], nonlinear optics [10, 11], high-dimensional quantum states [12–15], and high-capacity optical communications [16–22]. As the most representative OAM mode, the Laguerre–Gaussian (LG) beam has not only the topological charge *l*, but also the radial index of *p* (a non-negative integer), which is also very important both in theory and application [14, 23–26]. What’s more, as a vector field, the polarization *P* of a light beam is also a vital dimension except for *l* and *p*.

In this circumstance, the sorting of vector vortex beams (VVBs) according to the indexes of *l*, *p*, and *P* is a prerequisite operation in various applications mentioned above. When only one or two of the indexes are considered, it is not difficult to sort them. Recently, the sorting of angular index *l* has attracted much attention due to the infinite number of eigenstates, such as using optical geometric transformation [27–32], which uses less than two phase modulators. It is worth noting that metasurface can be used to sort both the polarization *P* and angular index *l* simultaneously [31]. However, it is not satisfactory for the radial index *p*, because *p* is mainly reflected in the intensity pattern, but not in the helical phase. The Gouy phase of LG mode is related to both *l* and *p*, which provides a potential method to sort *l* and *p* simultaneously [33–35]. The Gouy phase, however, is degenerate for the angular index of ±*l*, which means this method cannot sort *l* and −*l* directly. Alternatively, using the combination of Dove prisms and polarization beam splitters (PBSs) can sort *l* also [36], which, however, usually requires a system cascaded by many units. This means that the system’s complexity increases rapidly with its sorting capability, making it unsuitable for a larger number of mode sorting. Therefore, up to now, it is still a great challenge to sort all the three indexes with high efficiency, low crosstalk, and good extensibility, especially for a large number of modes.

It has been observed that mode sorting for the angular index *l* can be facilitated using two pure phase plates. Logically, employing more plates should enable the simultaneous sorting of both *l* and *p*. Following this rationale, the technology of multiplane light conversion (MPLC) has been introduced [37], which is widely used in mode multiplexing [38, 39] and mode conversion [40, 41]. In 2019, by arranging a Cartesian grid input layout, Fontaine et al. successfully sorted 210 LG modes [38]. However, this sorter requires an extra HG–LG mode converter, which undeniably compromises the integration of the device. Since 2018, Lin et al. have made significant strides with the introduction of diffractive deep neural network (D^2^NN) [42]. They leveraged the optical manipulation capabilities of MPLC and incorporated the gradient descent algorithm into the training process, which enhanced the performance of MPLC. D^2^NN has made great progress in image processing [43–52], and even in intelligent optical system [53–60]. In principle, with the use of polarization-dependent metasurface neural layers, D^2^NN can be employed to manipulate all photon dimensions, such as frequency, polarization, and spatial mode [32, 55]. Regrettably, aligning adjacent meta-layers (particularly in the visible spectrum) poses a significant challenge in practice. Additionally, the transmittance is notably low, which renders the scheme inefficient in applications.

In this study, we propose a vector vortex beam sorter based on D^2^NN both theoretically and experimentally. We successfully sort 120 modes (i.e., *l* ranges from −7 to 7, *p* from 0 to 3, and *P* for $\left|H\right\rangle$ and $\left|V\right\rangle$ ) in the visible spectrum. The proposed polarization D^2^NN, which eliminates the need for metasurface layers, consists of a single spatial light modulator (SLM), a mirror, and a polarization element. We anticipate that this efficient beam sorter will significantly advance the applications of vector vortex beams.

## 2 Results

### 2.1 Representation of vector vortex beams

Without loss of generality, the vector LG beams can be described in the basis of ( $\left|R\right\rangle$ , $\left|L\right\rangle$ ), or equally in the basis of ( $\left|H\right\rangle$ , $\left|V\right\rangle$ ),

(1) $$\begin{array}{cc} \left|\psi \right\rangle & =\underset{l,p}{\sum }\left(\alpha_{l,p}^{\text{R}}\left|{LG}_{l}^{p}\right\rangle \left|R\right\rangle +\beta_{l,p}^{\text{L}}\left|{LG}_{l}^{p}\right\rangle \left|L\right\rangle \right)\\ & =\underset{l,p}{\sum }\left(\alpha_{l,p}^{\text{H}}\left|{LG}_{l}^{p}\right\rangle \left|H\right\rangle +\beta_{l,p}^{\text{V}}\left|{LG}_{l}^{p}\right\rangle \left|V\right\rangle \right) \end{array},$$

where *α*_*l*,*p*_ and *β*_*l*,*p*_ are complex normalization coefficients. $\left|R\right\rangle$ , $\left|L\right\rangle$ , $\left|H\right\rangle$ , and $\left|V\right\rangle$ mean right circular, left circular, horizontal, and vertical polarization base vectors, respectively. $\left|{LG}_{l}^{p}\right\rangle$ is the LG mode, which is the earliest reported vortex beams carrying OAM and described as [1],

(2) $$\begin{array}{cc} LG_{l}^{p}\left(r,\varphi ,z\right) & =\frac{C_{l}^{p}}{\omega \left(z\right)}{\left(\frac{r\sqrt{2}}{\omega \left(z\right)}\right)}^{\left|l\right|}L_{p}^{\left|l\right|}\left(\frac{2r^{2}}{\omega {\left(z\right)}^{2}}\right)\\ & \;\times \exp\left(\frac{-r^{2}}{\omega {\left(z\right)}^{2}}\right)\exp\left(\frac{-\text{i}kr^{2}z}{2\left(z^{2}+z_{\text{R}}^{2}\right)}\right)\\ & \;\times \exp\left(\text{i}l\varphi +\text{i}\left(2p+|l|+1\right)\psi \left(z\right)\right) \end{array},$$

where *l* is the angular quantum number, and *p* is the radial quantum number. $C_{l}^{p}$ is a normalization constant and *k* is the wave number. The parameters of *ω*(*z*), *ψ*(*z*), and *z*_R_ are defined as,

(3) $$\begin{array}{cc} & \omega \left(z\right)=\omega_{0}\sqrt{1+{\left(\frac{z}{z_{\text{R}}}\right)}^{2}}\\ & \psi \left(z\right)=\left(2p+|l|+1\right)\arctan\frac{z}{z_{\text{R}}}\\ & z_{\text{R}}=\pi \omega_{0}^{2}/\lambda \end{array},$$

where *λ* is the wavelength and *ω*_0_ is the waist radius.

As the spatial light modulator (SLM) is typically designed solely for the $\left|H\right\rangle$ polarization, we conveniently employ the basis of ( $\left|H\right\rangle$ , $\left|V\right\rangle$ ) in the subsequent discussions. To successfully sort the $\left|V\right\rangle$ polarization, we initially separate the $\left|H\right\rangle$ and $\left|V\right\rangle$ modes using a polarizing beam splitter (PBS). The $\left|H\right\rangle$ polarization can then be directly input into the network from channel 1. As for the $\left|V\right\rangle$ polarization, we convert it into the $\left|H\right\rangle$ state using a polarization rotator before introducing it to the network from channel 2. In the end, the vector vortex modes are sorted through the D^2^NN from the two channels. By employing this method, we convert the polarization D^2^NN into two scalar D^2^NNs, which can be flexibly implemented using the widely available SLM’s in practice.

### 2.2 Scalar diffractive deep neural network

For the scalar diffractive deep neural network, according to the angular spectrum theory, the propagation of an optical field can be described as [61]

(4) $$\begin{array}{cc} & E\left(x,y,z\right)={\hat{F}}^{-1}\left\{H\left(k_{x},k_{y}\right)\hat{F}\left[E\left(x,y,0\right)\right]\right\}\\ & H\left(k_{x},k_{y}\right)=\exp\left(\text{i}z\sqrt{k^{2}-k_{x}^{2}-k_{y}^{2}}\right) \end{array},$$

where $\hat{F}$ is the operator for the two-dimensional Fourier transform, and correspondingly, ${\hat{F}}^{-1}$ represents the inverse Fourier transform. Here, *k* is the wavenumber of the light fields, and *k*_*x*_ and *k*_*y*_ are the *x* and *y* components, respectively.

**Figure 1:**
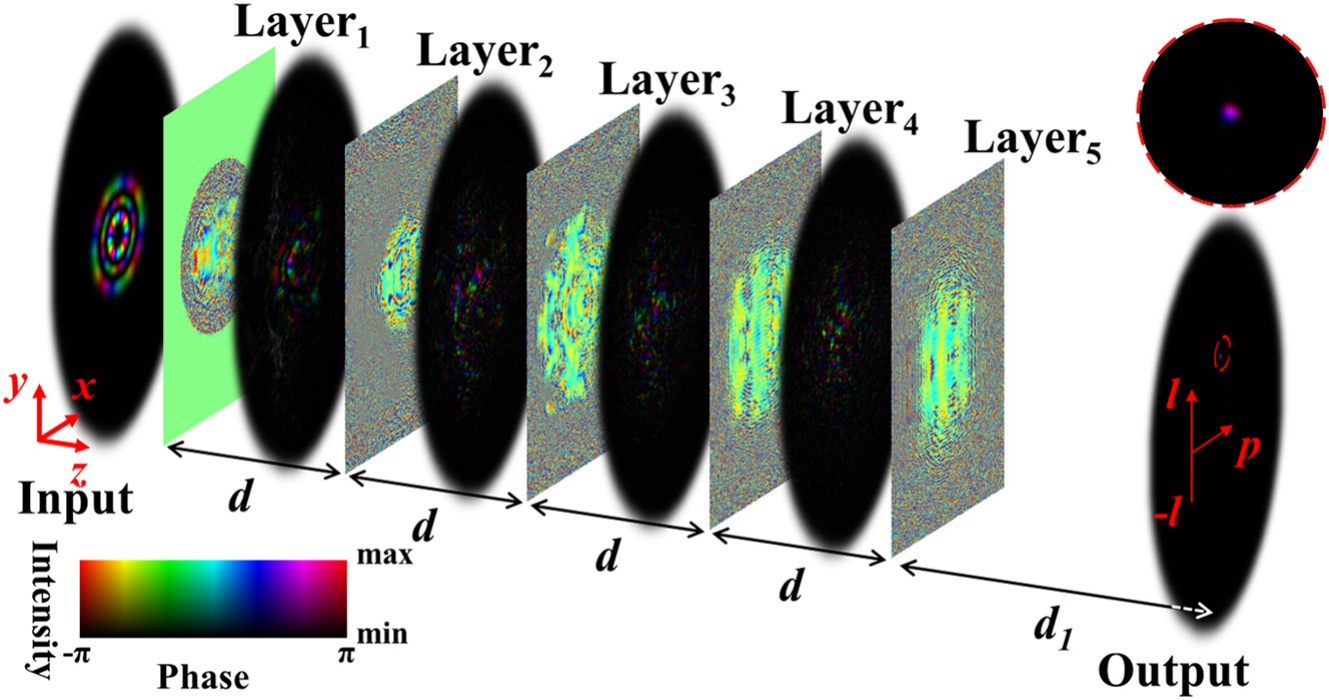
Schematic and numerical results of LG beam sorting according to the angular index *l*, radius index *p*, and polarization *P* using the proposed D^2^NN, which is physically formed by five diffraction layers of Layer_1,2,3,4,5_. The distances between two neighboring layers *d* is 3.5 cm, and the distance from the last layer to the output plane *d*_1_ is 9.3 cm. For clarity, the upper right insert (red circle) shows the zoom in view of the output beam.

Backward propagating and gradient descent algorithms are used to train the D^2^NN. For the training set of LG beams, angular index *l* is from −7 to 7, and radial index *p* is from 0 to 3 (a total number of 60 modes for one orthogonal polarization). Without loss of generality, five neural layers are set in for the D^2^NN, and each layer has 310 × 420 optical neurons. The neuron size is set to be 8 μm × 8 μm to match the pixel size of SLM’s in the experiment (as shown in the following section). The wavelength *λ* of incident light is 532 nm. The distances between two neighboring layers *d* are set to be 3.5 cm, and the distance from the last layer to the output plane *d*_1_ is 9.3 cm, as illustrated in Figure 1. More details about the training are shown in Section I of Supplementary Material.

Figure 1 shows the schematic structure and a typical numerical result of mode sorting of the D^2^NN after training. When propagating through the five diffraction layers (Layers_1,2,3,4,5_), the input LG mode (input) is directed to a pre-defined position on the output plane (output), which is co-determined by the parameters of *l*, *p*, and polarization state *P*. The accompanying video provides further details regarding beam propagation.

**Video 1:** 

### 2.3 Experimental details

**Figure 2:**
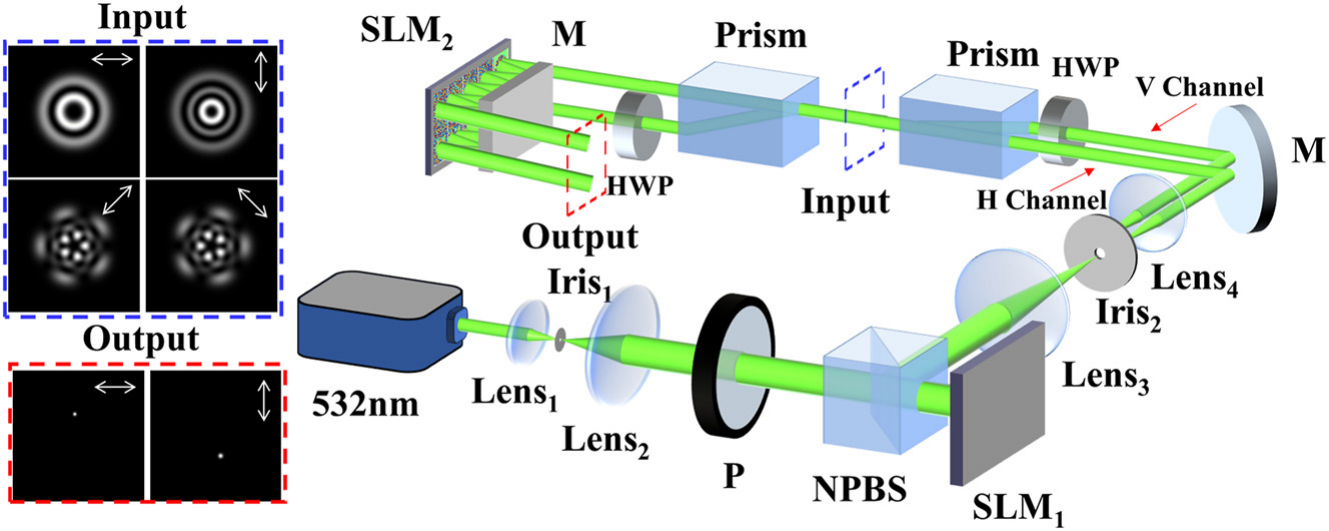
The experimental setup for sorting vector vortex beams using the D^2^NN. A Gaussian laser beam with a wavelength of 532 nm is expanded and collimated by Lens_1,2_ and the pinhole Iris_1_. This is followed by a polarizer (*P*), which aligns the polarization along the horizontal direction to match SLM_1_. SLM_1_ then transforms the Gaussian beam into one or more LG beams with varying *l* and *p* values (two LG beams are illustrated as an example in this figure). After passing through the 4-f system, the polarization of one beam is switched from $\left|H\right\rangle$ to $\left|V\right\rangle$ by the half-wave plate (HWP). A calcite crystal is then used to combine the two orthogonally polarized beams into one, which serves as the incident vector vortex beam to be sorted. Finally, the input vector vortex beams are sorted by the D^2^NN, physically formed by a calcite crystal, HWP, SLM_2_, and a mirror (M). The upper left inset (enclosed by the blue dashed square) displays the intensity patterns of the input vector vortex beam, checked using a linear polarizer along 0°, 90°, 45°, and 135° directions. The lower left inset (enclosed by the red dashed square) shows the output of the mode sorting from the channels of $\left|H\right\rangle$ and $\left|V\right\rangle$ . The abbreviations used are: non-polarizing beam splitter (NPBS); spatial light modulator (SLM); half-wave plate (HWP); mirror (M); and polarizer (*P*).

In order to verify the performances of the vector vortex beam sorter in practice, we built an experimental system, as shown in Figure 2. In the system, the first part from the “532 nm” laser to the “input” plane is for the preparation of the LG beams with various *l*, *p*, and polarization *P*. The rest part is the D^2^NN. The incident Gaussian laser beam with a wavelength of 532 nm is expanded and collimated by Lens_1_, Lens_2_, and the pinhole Iris_1_. Using the polarizer P, the polarization of the incident beam is forced into $\left|H\right\rangle$ state because commercial SLM’s are calibrated for horizontal polarization only. Subsequently, LG beams with varying *l* and *p* are generated using a forked phase hologram displayed on SLM_1_ (Holoeye PLUTO-2 VIS016) as detailed in Section III of the Supplementary Material. Through the 4f system (Lend_3_, Lend_4_, and Iris_2_), we obtain the first order diffracted beam consisting of two LG beams, each corresponding to a distinct orthogonal polarization channel. The polarization of one of these beams is transitioned from $\left|H\right\rangle$ to $\left|V\right\rangle$ by a half-wave plate (HWP). Finally, using a calcite crystal, the two orthogonally polarized LG beams are re-combined into one beam again, which mimics the input beam, which is the superposition of multiple LG beams with different *l*, *p*, and polarization *P* for sorting. The upper left insert shows the intensity patterns of the input beam checked by a polarizer along different directions.

In the case of sorting, we firstly use a calcite crystal and an HWP to separate the two orthogonal polarizations of $\left|H\right\rangle$ and $\left|V\right\rangle$ . The $\left|H\right\rangle$ component is sent into the upper D^2^NN directly for sorting (here, we name it as the $\left|H\right\rangle$ channel for convenience). For the $\left|V\right\rangle$ component, an HWP is used to transform the $\left|V\right\rangle$ into $\left|H\right\rangle$ state firstly, because the SLM’s can modulate the $\left|H\right\rangle$ polarization only. Then, it is sent into the lower D^2^NNs for sorting (the $\left|V\right\rangle$ channel). It is noted that both the D^2^NNs of $\left|H\right\rangle$ and $\left|V\right\rangle$ channels are physically formed by SLM_2_ (Holoeye PLUTO-2 NIR011) and the mirror M. Here, the layer of the network is set to be 5, and SLM_2_ is divided into 10 regions (more details are shown in Section II of Supplementary Material). Using this configuration, one SLM is enough to realize the two 5-layer D^2^NNs physically used in Figure 1, which is very convenient in experiments. The lower left insert shows the sorting result on the output plane. Clearly, different LG modes are separated completely.

## 3 Discussion and conclusions

**Figure 3:**
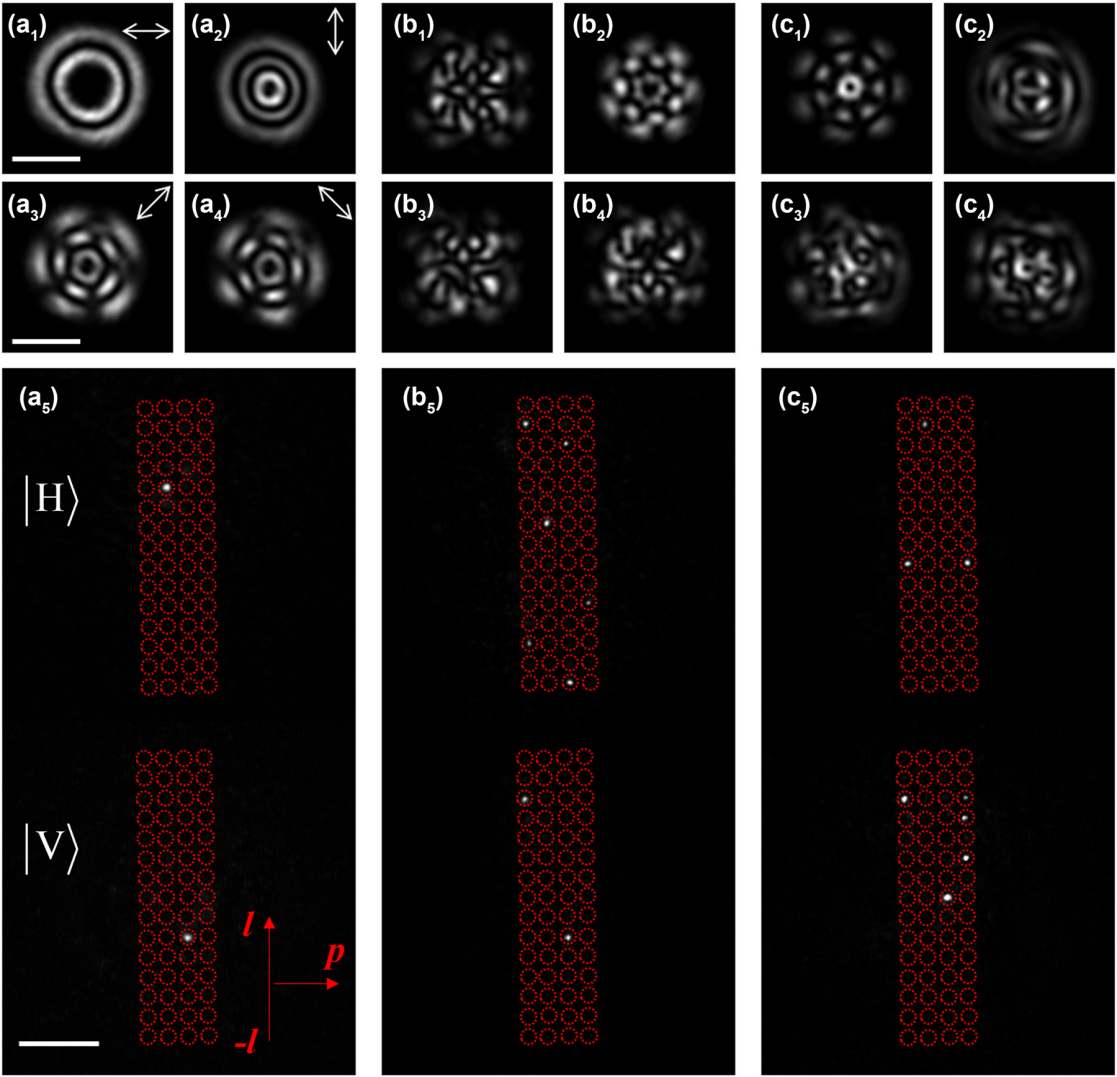
Experimental results of the vector vortex beam sorter in different cases of input. (a_1_–a_5_) are for the input of $\left|\psi_{\text{in}}\right\rangle =\left|{LG}_{3}^{1}\right\rangle \left|H\right\rangle +\left|{LG}_{-2}^{2}\right\rangle \left|V\right\rangle$ . (a_1_–a_4_) are the intensity patterns of the input beam after a linear polarizer aligned at 0°, 90°, 45°, and 135°, respectively. (a_5_) is the sorting result on the output plane. The dashed circles mark the output positions (which are pre-defined in the network training) of different LG modes according to *P*, *l*, and *p*. The upper half is for $\left|H\right\rangle$ and the lower half is for $\left|V\right\rangle$ . From bottom to top, *l* increases from −7 to 7, and from left to right, *p* increases from 0 to 3. Clearly, at the positions of $\left|{LG}_{3}^{1}\right\rangle \left|H\right\rangle$ and $\left|{LG}_{-2}^{2}\right\rangle \left|V\right\rangle$ , two bright dots are observed, which show the correctness of the sorting. (b_1_–b_5_) are the same as (a_1_–a_5_), except for $\left|\psi_{\text{in}}\right\rangle =\left(\left|{LG}_{6}^{0}\right\rangle +\left|{LG}_{5}^{2}\right\rangle +\left|{LG}_{1}^{1}\right\rangle +\left|{LG}_{-3}^{3}\right\rangle +\left|{LG}_{-5}^{0}\right\rangle +\left|{LG}_{-7}^{2}\right\rangle \right)\left|H\right\rangle +\left(\left|{LG}_{5}^{0}\right\rangle +\left|{LG}_{2}^{2}\right\rangle \right)\left|V\right\rangle$ . (c_1_–c_5_) are the same as (a_1_–a_5_), except for $\left|\psi_{\text{in}}\right\rangle =\left(\left|{LG}_{6}^{1}\right\rangle +\left|{LG}_{-1}^{0}\right\rangle +\left|{LG}_{-1}^{3}\right\rangle \right)\left|H\right\rangle +\left(\left|{LG}_{5}^{0}\right\rangle +\left|{LG}_{5}^{3}\right\rangle +\left|{LG}_{4}^{3}\right\rangle +\left|{LG}_{2}^{3}\right\rangle +\left|{LG}_{0}^{2}\right\rangle \right)\left|V\right\rangle$ (scale bar: 500 μm).

Figure 3 shows the experimental results in cases of various incident LG modes. The upper two rows show the intensity patterns of the input beams after a polarization analyzer aligned along 0°, 90°, 45°, and 135°. Clearly, the incident beam can be a very complicated superposition of multiple LG beams with different *l*, *p*, and polarization *P*.

The bottom row of Figure 3 shows the sorting results on the output plane, where the upper group is for the |H⟩ channel, and the lower group is for the |V⟩ channel. Here, the angular index *l* increases from −7 to 7 (from bottom to top), and the radial index *p* increases from 0 to 3 (from left to right). Therefore, the total number of LG modes that can be sorted in the current experiment setup is 120. All the positions are highlighted by the red circles for clarity. Figure 3(a_1_–a_5_) is for the incident beam of $|\psi_{\text{in}}〉=|{LG}_{3}^{1}〉|H〉+|{LG}_{-2}^{2}〉|V〉$ , and the sorting result is satisfactory. When the superposition of eight different LG modes are incident simultaneously, the sorting results are also satisfactory, as shown in Figure 3(b_1_–b_5_) where $|\psi_{\text{in}}〉=\left(|{LG}_{6}^{0}〉+|{LG}_{5}^{2}〉+|{LG}_{1}^{1}〉+|{LG}_{-3}^{3}〉+|{LG}_{-5}^{0}〉+|{LG}_{-7}^{2}〉\right)|H〉+\left(|{LG}_{5}^{0}〉+|{LG}_{2}^{2}〉\right)|V〉$ , and Figure 3(c_1_–c_5_) where $|\psi_{\text{in}}〉=\left(|{LG}_{6}^{1}〉+|{LG}_{-1}^{0}〉+|{LG}_{-1}^{3}〉\right)|H〉+\left(|{LG}_{5}^{0}〉+|{LG}_{5}^{3}〉+|{LG}_{4}^{3}〉+\right.\left.|{LG}_{2}^{3}〉+|{LG}_{0}^{2}〉\right)|V〉$ .

**Figure 4:**
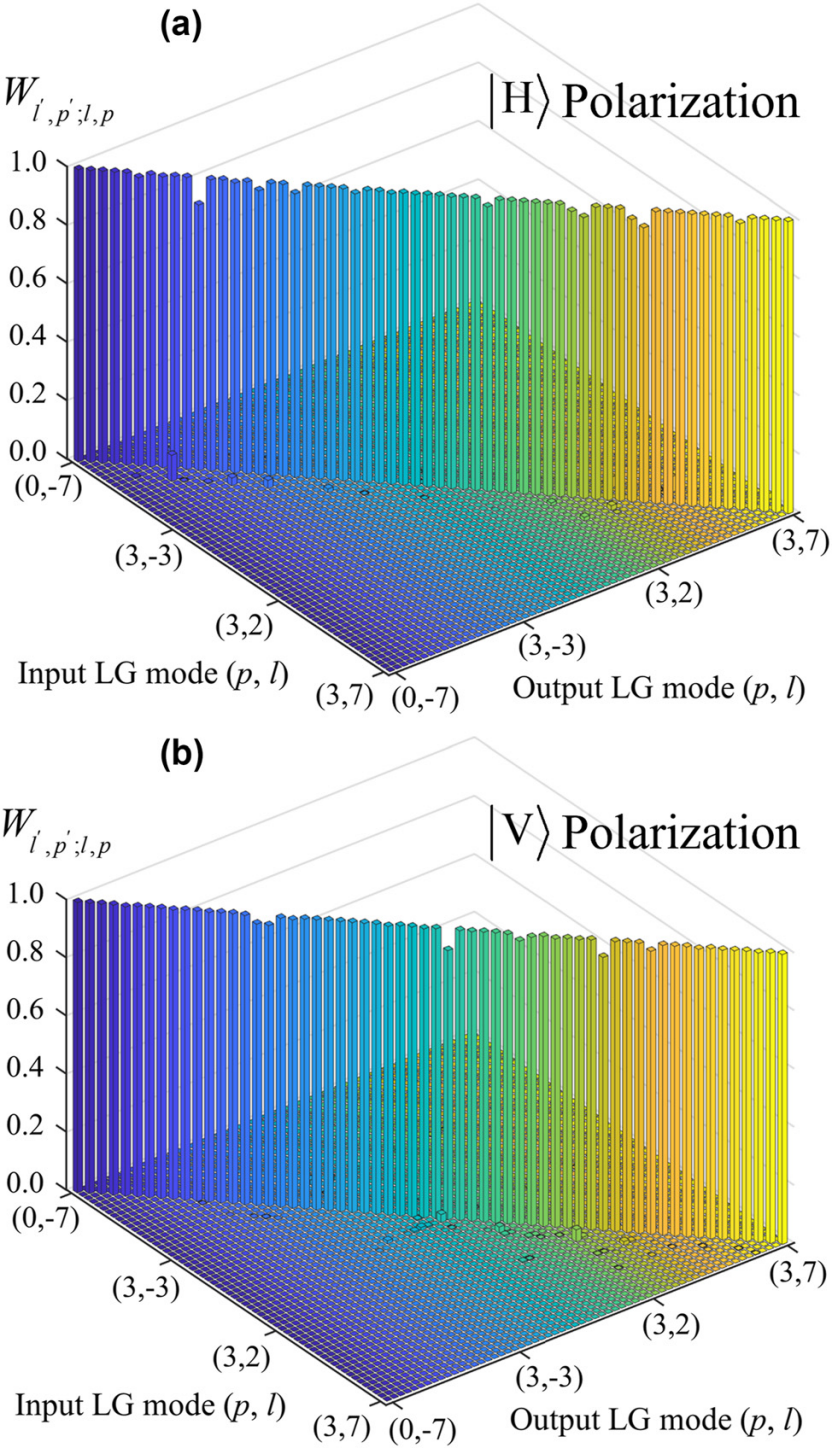
Normalized energy *W*_*l*′,*p*′;*l*,*p*_ of the sorter at the output plane, which is the measured energy of mode ${LG}_{l^{\prime }}^{p^{\prime }}$ normalized by the total energy of output in case of incident $\left|\psi_{\text{in}}\right\rangle ={LG}_{l}^{p}$ . Here *l* and *l*′ change from −7 to 7, and *p* and *p*′ change from 0 to 3. All the parameters are the same as those in Figure 3. (a) is for $\left|H\right\rangle$ polarization, and (b) is for $\left|V\right\rangle$ polarization.

From Figure 3, one can quantitatively find the effectiveness of the D^2^NN mode sorter. Here, we measure the performances of the mode sorter using the normalized output energy of *W*_*l*′,*p*′;*l*,*p*_, which is the measured energy of L $G_{l^{\prime }}^{p^{\prime }}$ mode normalized by the total output energy in case of the incident of L $G_{l}^{p}$ mode, and the results for both polarizations are shown in Figure 4 (all the other parameters are the same as those in Figure 3). The diagonal elements of *W*_*l*′,*p*′;*l*,*p*_ can be regarded as the energy efficiencies for each LG mode, and the off-diagonal elements show the crosstalk between different modes.

Figure 4 shows that the energy efficiencies of the sorting are high in the output plane, and the average value of *E*_*l*,*p*;*l*,*p*_ is about 99.43 %. The average crosstalk of *W*_*l*′,*p*′;*l*,*p*_ with *l* ≠ *l*′ and *p* ≠ *p*′ is near 0 % for most cases, and the maximum crosstalk does not exceed −12 dB. Those merits of high energy efficiency and low crosstalk are vital for the practical applications of vector vortex beams. Here, we also note that the existing crosstalk in the experiment can be attributed to the aberrations accumulated when passing through the SLM’s. In principle, these errors can be greatly suppressed, such as using the Zernike function-based phase compensation [45, 46, 57]. In reality, for this system, the primary source of loss is the SLM, which has a reflectivity of approximately 67 %. Consequently, for a 5-layer D^2^NN, the transmittance does not exceed 15 % (−8.2 dB). Utilizing an SLM with a dielectric mirror (with a high reflectivity above 95 %) can increase the total transmittance to 77 % (−1.1 dB). And the average efficiency of the system is about 57 % (−5.6 dB) in simulation. On the other hand, we currently use cross entropy as the loss function to achieve low crosstalk. When the aim is focused on efficiency enhancement, the loss function can be adjusted accordingly, and this flexibility is one of the key advantages of the D^2^NN framework.

Although the mode sorter is designed for a specific wavelength, it carries the potential for efficient operation across a broad bandwidth [38, 39]. A shift in the incident wavelength from the optimized value will result in deviations in the phase modulations on each diffraction layer. As detailed in Section IV of the Supplementary Material, the full width at half maximum (FWHM) is about 5 nm at the optimized wavelength of 532 nm. However, when the optimized wavelength is 1550 nm, the FWHM bandwidth expands to approximately 20 nm. Interestingly, if bandwidth considerations are integrated into the training of D^2^NN, it is feasible to further increase the bandwidth, as discussed in Section IV of the Supplementary Material.

In summary, we have introduced an efficient vector vortex beam (VVB) sorter that manages all the three degrees of freedom, namely the polarization (*P*), the angular index (*l*), and the radial index (*p*) of LG beams, utilizing the diffractive deep neural network (D^2^NN). Experimental results in the visible spectrum at 532 nm demonstrate that our mode sorter can efficiently handle up to 120 modes, maintaining a low crosstalk of less than −12 dB. For optical communication applications, large-scale mode multiplexing could notably augment system capabilities. We believe our VVB sorter will prove to be an invaluable tool in practical applications of multidimensional multiplexed optical communication and high-dimensional quantum information systems. Moreover, it offers a versatile and universal approach to polarization D^2^NNs.

## Supplementary Material

Supplementary Material Details

Supplementary Material Details
