# Supplementary material for: Vector vortex beams sorting of 120 modes in visible spectrum
Source: Nanophotonics. 2023 Oct 4;12(20):3955–62. doi: 10.1515/nanoph-2023-0482 (PMC11501642; doi:10.1515/nanoph-2023-0482)
Supplement: Supplementary file 3 — Supplementary Material Details [file j_nanoph-2023-0482_suppl_001.pdf]

Supplementary Materials for

Vector vortex beams sorting of 120 modes in visible spectrum

Qi Jia,[1] Yanxia Zhang,[1] Bojian Shi,[1] Hang Li,[1] Xiaoxin Li,[1] Rui Feng,[1] Fangkui Sun,[1] Yongyin Cao,[1] Jian Wang,[2,*] Cheng-Wei Qiu,[3,†] and Weiqiang Ding[1,4,‡]

[1]*Institute of Advanced Photonics, School of Physics, Harbin Institute of Technology, Harbin 150001, China*
[2]*School of Physics, Harbin Institute of Technology, Harbin 150001, China*
[3]*Department of Electrical and Computer Engineering, National University of Singapore, Singapore 117583, Singapore*
[4]*Collaborative Innovation Center of Extreme Optics, Shanxi University, Taiyuan 030006, Shanxi, China*

(Dated: September 10, 2023)

This supplementary materials provide the design details about the D^2NN for vector Laguerre-Gaussian (LG) mode sorter, which is constructed based on two scalar D^2NN's for horizontally polarized LG modes. We also provide a brief overview of the hologram used for generating LG beams and discuss the bandwidth of this system.

CONTENTS

I. D^2NN for horizontally polarized LG modes 2
II. Vector LG mode sorter 3
III. The hologram of generating LG beam 4
IV. The bandwidth of the sorting system 5
References 6

* hitwj@hit.edu.cn
† chengwei.qiu@nus.edu.sg
‡ wqding@hit.edu.cn

I. D^{2}NN FOR HORIZONTALLY POLARIZED LG MODES

As shown in the main text, we use D^{2}NN to sort the LG modes, which is based on the propagation of LG mode through several layers (5 layers in the main text) of diffraction plane. According to the principle of Fourier optics, the propagation of an optical field can be described as [1],

$$\begin{cases} E(x,y,z) = \hat{\mathcal{F}}^{-1}\left\{\hat{\mathcal{H}}\hat{\mathcal{F}}\left[E(x,y,0)\right]\right\} \\ \hat{\mathcal{H}} = \exp\left(iz\sqrt{k^2-k_x^2-k_y^2}\right) \end{cases}, \tag{S1}$$

where $\hat{\mathcal{F}}$ is the operator for the 2D Fourier transform, and correspondingly, $\hat{\mathcal{F}}^{-1}$ represents the inverse 2D Fourier transform. $\hat{\mathcal{H}}$ is the transfer function. k is the angular wavenumber of the light fields, and k_x and k_y are the projections along x and y directions, respectively.

In the case of finite sampling, the integral in Equation (S1) can be rearranged into the form of the matrix, which is

$$E(x,y,z) = \mathcal{F}^{-1}\mathcal{H}\mathcal{F}E(x,y,0), \tag{S2}$$

where $\mathcal{F}$ is the 2D discrete Fourier transform, and correspondingly, $\mathcal{F}^{-1}$ represents the inverse 2D discrete Fourier transform. $\mathcal{H}$ is the discrete transfer function. For convenience, we define the diffractive matrix D as

$$D = \mathcal{F}^{-1}\mathcal{H}\mathcal{F}. \tag{S3}$$

For the building of the D^{2}NN, we use Equations (S2 & S3) to simulate the forward propagation,

$$\begin{cases} X_{n+1}^{(\alpha)} = DT_nX_n^{(\alpha)} \\ T_n = \mathrm{diag}(\exp\left(j\varphi_n\right)) \end{cases}, \tag{S4}$$

where $X_n^{(\alpha)}$ is a column vector of the input light on the $n\text{-}th$ diffractive layer for the $\alpha\text{-}th$ sample. φ_n is a column vector of the phase in $n\text{-}th$ diffractive layer. $\mathrm{diag}(\cdot)$ represents the diagonalization operation. The total number of diffractive layers is set to be N.

To reduce the cross-talking of the modes, we use the fidelity (F) to conduct the cross entropy as the loss function for the diffractive deep neural network,

$$\begin{cases} \mathsf{F}^{(\alpha,\beta)} = \left|Y_{tar}^{(\alpha)\dagger}X_{out}^{(\beta)}\right|^2 \\ \mathsf{Q}^{(\alpha,\beta)} = \delta_{(\alpha,\beta)} \\ \mathcal{L} = -\dfrac{1}{Num}\displaystyle\sum_{\alpha,\beta}\left[\mathsf{Q}^{(\alpha,\beta)}\lg\left(\mathsf{F}^{(\alpha,\beta)}\right) + \left(1-\mathsf{Q}^{(\alpha,\beta)}\right)\lg\left(1-\mathsf{F}^{(\alpha,\beta)}\right)\right] \end{cases}, \tag{S5}$$

where $Y_{tar}^{(\alpha)}$ and $X_{out}^{(\alpha)}$ are the target output and the actual output of the D^{2}NN for $\alpha\text{-}th$ sample, respectively. The total number of training samples is Num. $\mathsf{Q}^{(\alpha,\beta)}$ is the target fidelity for the $\alpha\text{-}th$ target output and $\beta\text{-}th$ input mode. $\delta_{(\alpha,\beta)}$ is the Dirac delta function. In the training, the gradient of the loss function $\mathcal{L}$ with respect to φ_n is the most important item to be calculated. According to Equation (S5), the derivative of $\mathcal{L}$ with respect to φ_n is

$$\begin{cases} \dfrac{\partial\mathcal{L}}{\partial\varphi_n} = -\dfrac{1}{Num}\displaystyle\sum_{\alpha,\beta}K^{(\alpha,\beta)}\left(Y_{tar}^{(\alpha)\dagger}X_{out}^{(\beta)}\right)^*Y_{tar}^{(\alpha)\dagger}\dfrac{\partial X_{out}^{(\beta)}}{\partial\varphi_n} + c.c. \\ \dfrac{\partial X_{out}^{(\beta)}}{\partial\varphi_n} = DT_NDT_{N-1}\cdots\dfrac{\partial DT_nX_n^{(\beta)}}{\partial\varphi_n} \\ K^{(\alpha,\beta)} = \dfrac{\mathsf{Q}^{(\alpha,\beta)}}{\mathsf{F}^{(\alpha,\beta)}} - \dfrac{1-\mathsf{Q}^{(\alpha,\beta)}}{1-\mathsf{F}^{(\alpha,\beta)}} \end{cases}. \tag{S6}$$

In order to facilitate the calculation, we further simplify Equation (S6) and get Equation (S7) [2], which is

$$\frac{\partial\mathcal{L}}{\partial\varphi_n} = -\frac{1}{Num}\sum_{\alpha,\beta}\left[P_n^{(\alpha,\beta)}\right]^T\frac{\partial T_n}{\partial\varphi_n}diag\left(X_n^{(\beta)}\right) + c.c., \tag{S7}$$

with

$$P_n^{(\alpha,\beta)} = D^T \prod_{\gamma=N}^{n+1} \left(T_\gamma D^T\right) \left[K^{(\alpha,\beta)} \left(Y_{tar}^{(\alpha)\dagger} X_{out}^{(\beta)}\right)^* Y_{tar}^{(\alpha)*}\right]. \tag{S8}$$

In Equation (S7), we divide $\frac{\partial \mathcal{L}}{\partial \varphi_n}$ into 3 parts, $P_n^{(\alpha,\beta)}$, $\frac{\partial T_n}{\partial \varphi_n}$ and $X_n^{(\beta)}$. $P_n^{(\alpha,\beta)}$ represents the backward propagation part, $X_n^{(\beta)}$ represents the frontward propagation part.

According to Equations (S4, S7 & S8), we train the D^{2}NN for the target of LG mode sorting. The training set includes LG modes with various l and p as the input and Gaussian spots with various central positions as the target output. Here, the angular quantum index l changes from -7 to 7, and the radius quantum index p changes from 0 to 3 (the total number of modes is $15 \times 4 = 60$). The total number of neural layers is $N = 5$, and each layer has 310×420 optical neurons with the neuron size of 8×8 μm (matching the pixel size of SLM used in the experiment), and the wavelength λ is in the visible spectrum of 532 nm. Moreover, the distance between neighboring layers d is 3.51 cm, and the distance from the last layer to the output plane d_1 is 9.3 cm. Specifically, we set the learning rate at 0.03 and maintained a batch size of 60, consistent with Num, and used the Adam optimizer with a momentum coefficient of (0.9, 0.999).

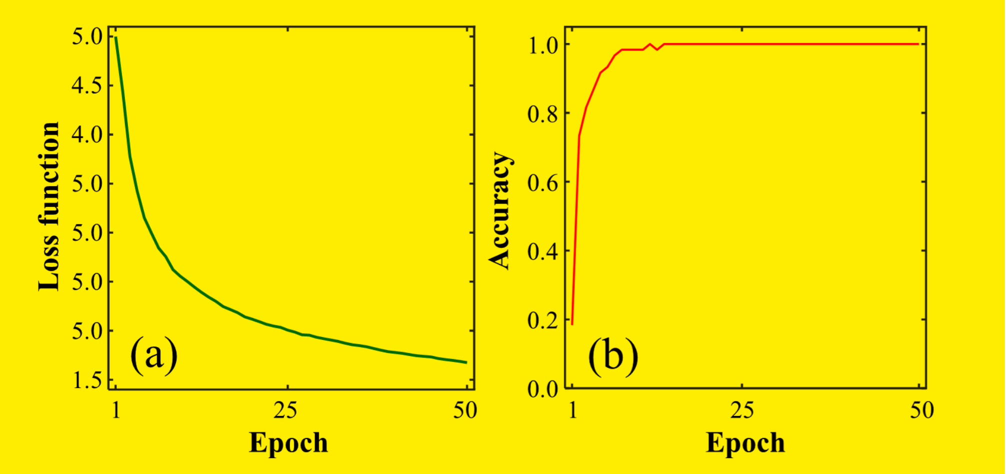

Figure S1. Numerical results of the loss function and sorting accuracy of the 5-layer D^{2}NN.

To ensure that the input LG mode is accurately sorted into its corresponding channel, we introduce a metric M_α for measurement. The overall accuracy M_α is then given by $\mathsf{M} = \frac{1}{Num}\sum_\alpha \mathsf{M}_\alpha$, where M_α is defined as follows,

$$\mathsf{M}_\alpha = \begin{cases} 1 & \text{when} \quad \alpha = \text{argmax}\left\{\mathsf{F}^{(\alpha,\beta)}\right\} \\ 0 & \text{when} \quad \alpha \neq \text{argmax}\left\{\mathsf{F}^{(\alpha,\beta)}\right\} \end{cases}, \tag{S9}$$

where $\mathsf{F}^{(\alpha,\beta)}$ represents the fidelity between samples α-th and β-th. The function argmax identifies the index at which F reaches its maximum value as β varies from 1 to Num.

After training, the D^{2}NN can be used to sort LG mode, and the results are shown in Figure S1. There, the numerical simulation of the LG sorter is implemented on the platform of Python version 3.9.12 with Pytorch framework version 1.11.0 (Meta. Inc.) using a desktop computer (Nvidia GeForce 2080 Super Graphical Processing Unit, GPU, and Intel Core i7 12700 CPU at 2.10 GHz with 12 cores, 32 GB of RAM, running with a Microsoft Windows 10 operating system).

II. VECTOR LG MODE SORTER

As shown in the main text, the vector LG mode can be divided into 2 parts of $|\mathrm{H}\rangle$ and $|\mathrm{V}\rangle$,

$$|\psi\rangle = \sum_{l,p} \left(\alpha_l^p \,|\mathrm{LG}_l^p\rangle\, |\mathrm{H}\rangle + \beta_l^p \,|\mathrm{LG}_l^p\rangle\, |\mathrm{V}\rangle\right). \tag{S10}$$

The operator of vector vortex beam sorter ($\hat{O}_{VSB}$) can be written as,

$$\begin{aligned}\hat{O}_{VSB} &= \hat{O}_{LG}^{(H)} \left|\mathrm{H}\right\rangle \left\langle\mathrm{H}\right| + \hat{O}_{LG}^{(V)} \left|\mathrm{V}\right\rangle \left\langle\mathrm{V}\right| \\ &= \begin{bmatrix} \hat{O}_{LG}^{(H)} & 0 \\ 0 & \hat{O}_{LG}^{(V)} \end{bmatrix} \end{aligned}, \tag{S11}$$

where $\hat{O}_{LG}^{(H)}$ and $\hat{O}_{LG}^{(V)}$ are the scalar LG mode sorter, which can be designed according to the details presented in the previous section. However, most available commercial liquid-crystal Spatial Light Modulators (LC-SLM) can only modulate for horizontal polarization. The Jones matrix of LC-SLM can be written as,

$$\hat{J}_{LC-SLM} = \begin{bmatrix} \exp(j\varphi(x,y)) & 0 \\ 0 & 1 \end{bmatrix}. \tag{S12}$$

According to Equation (S11), $\hat{O}_{VSB}$ is a diagonal matrix. Thus, we can firstly use the polarization beam splitter (PBS) to separate the two orthogonal polarizations and transform the $\left|\mathrm{V}\right\rangle$ to $\left|\mathrm{H}\right\rangle$ by a half wave plate (HWP), and then use the SLM to modulate the two beams separately. This process can be expressed more clearly as,

$$\begin{aligned}\hat{O} &\to \hat{P}^{(H)} \left|\mathrm{H}\right\rangle \left\langle\mathrm{H}\right| + \hat{P}^{(V)} \left|\mathrm{V}\right\rangle \left\langle\mathrm{V}\right| && (PBS) \\ &\to \hat{P}^{(H)} \left|\mathrm{H}\right\rangle_1 \left\langle\mathrm{H}\right| + \hat{P}^{(V)} \left|\mathrm{H}\right\rangle_2 \left\langle\mathrm{V}\right| && (HWP) \\ &\to \hat{O}_{SLM}^{(H)} \left|Channel_1\right\rangle \left\langle\mathrm{H}\right| + \hat{O}_{SLM}^{(V)} \left|Channel_2\right\rangle \left\langle\mathrm{V}\right| && (SLM)\end{aligned} \tag{S13}$$

where $\hat{P}^{(H)}$ and $\hat{P}^{(V)}$ represent the operator of PBS for $\left|\mathrm{H}\right\rangle$ and $\left|\mathrm{V}\right\rangle$, respectively. $\hat{O}_{SLM}^{(H)}$ and $\hat{O}_{SLM}^{(V)}$ represent the operators of SLM for the beams from the two polarization channels.

As shown in Equations (S11 & S13), we need to replace $\hat{O}_{SLM}$ with $\hat{O}_{LG}$ to achieve vector vortex beam sorter, which is shown in Figure S2.

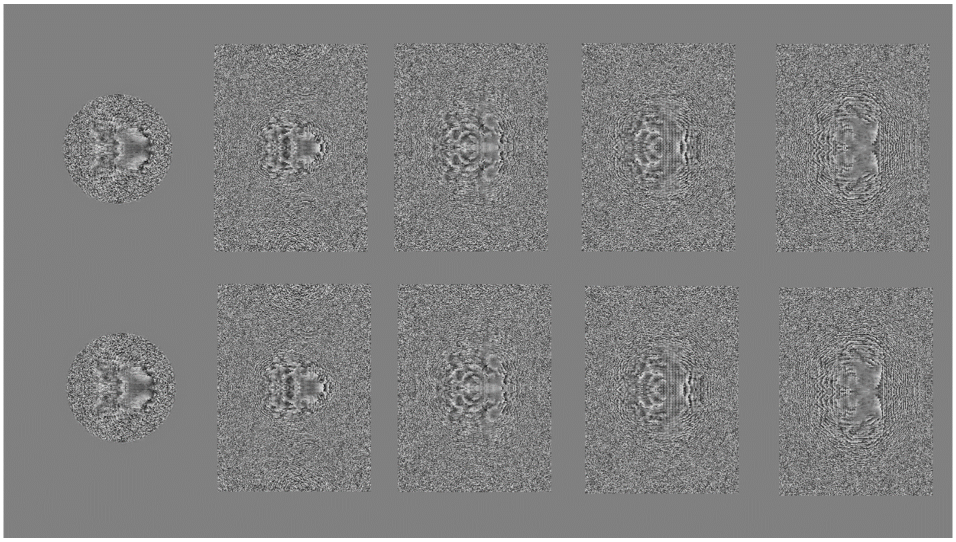

Figure S2. The phase pattern loaded on SLM is calculated according to the D^{2}NN.

In a nutshell, we use the diffractive deep neural network with a SLM and other common optical elements to achieve the vector vortex beam sorter, according to Equations (S4, S7,S8, S11 & S13).

III. THE HOLOGRAM OF GENERATING LG BEAM

In our experiment, we employ a SLM to simultaneously generate different (LG) modes in the horizontal ($\left|\mathrm{H}\right\rangle$) and vertical ($\left|\mathrm{V}\right\rangle$) polarization channels. According to Refs [3, 4], we use Equation (S14) to calculate hologram in order to get the desired output beam ($E = Ae^{j\Phi}$),

$$\begin{aligned}\mathcal{M} &= 1 + \frac{1}{\pi}\,\mathrm{sinc}^{-1}(A)\\ \mathcal{F} &= \Phi - \pi\mathcal{M}\\ \mathrm{T} &= \exp(j\mathcal{M}\mathcal{F})\end{aligned}, \qquad \text{(S14)}$$

where A and Φ represent the amplitude and phase of the desired output beam, respectively. $\mathrm{sinc}^{-1}(\cdot)$ is the inverse function of the sinc, and $\mathrm{sinc}(x) = \sin(x)/x$ is an unnormalized sinc function in the domain of $[-\pi, 0]$. And T is the calculated hologram. In Figure S3, we show the hologram calculated according to Equation (S14).

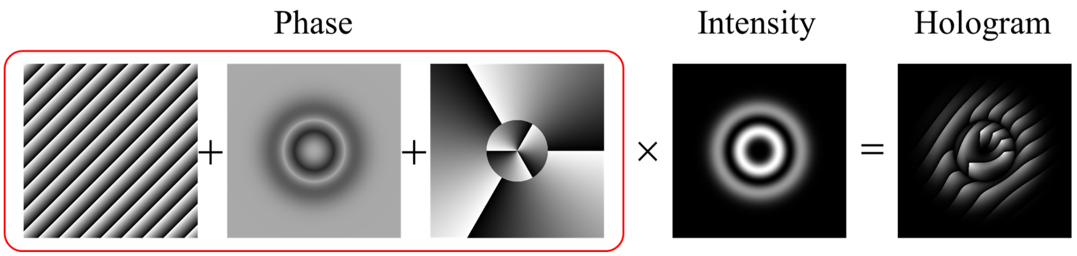

Figure S3. Calculated hologram used to generate the designated LG beam.

To generate vector vortex beams, we create two Laguerre-Gaussian (LG) beams corresponding to orthogonal polarizations, simultaneously. The final hologram loaded on SLM_1 is depicted in Figure S4. This figure displays two channels, each corresponding to one of the orthogonal polarizations. The distance d_0 represents the spatial separation between these polarizations, induced by a calcite crystal. In our setup, d_0 is about 3.8 mm. The focused distances for Lens_3 and Lens_4 are 20 and 10 cm, respectively, indicating that the size of the beam passing through Lens_3 and Lens_4 is reduced by a factor of 0.5. Thus, the distances of two beams in hologram is $2\mathrm{d}_0$.

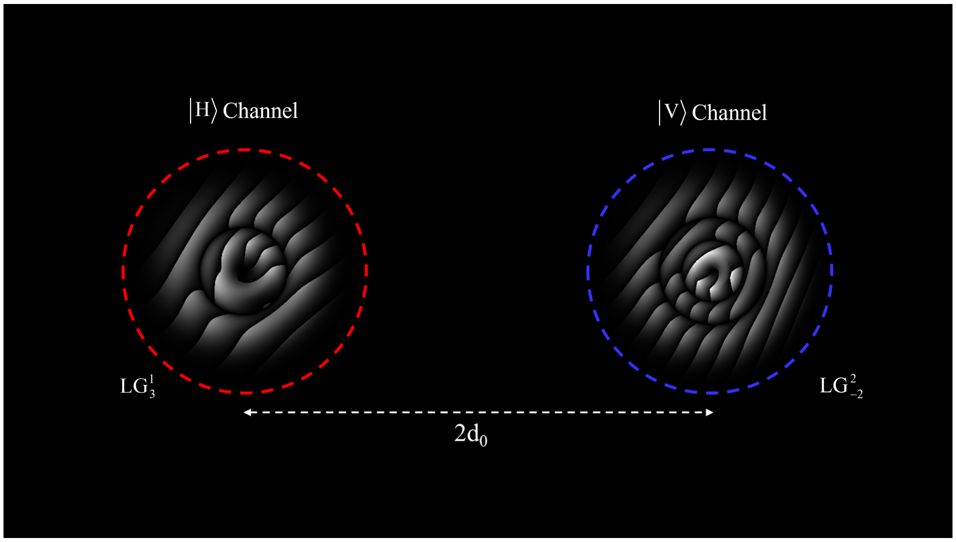

Figure S4. The hologram loaded onto SLM_1 generates the input state $|\psi_{in}\rangle = |\mathrm{LG}_3^1\rangle\,|\mathrm{H}\rangle + |\mathrm{LG}_{-2}^2\rangle\,|\mathrm{V}\rangle$.

IV. THE BANDWIDTH OF THE SORTING SYSTEM

Bandwidth is invariably a critical parameter within an optical system. In the context of our simulation, we further delve into the bandwidth of the sorting system. For simplicity, we disregard the dispersion of liquid crystals, which implies that the phase difference is primarily dependent on the wavelength, and discuss the bandwidth of the sorting system in the simulation. This assumption holds true particularly when the variation in wavelength is

small. Consequently, for a light wavelength denoted as λ, the phase plane is defined as $T = \exp\left(j\varphi\frac{\lambda_0}{\lambda}\right)$, where φ represents the phase of the diffractive layer and λ_0 is the optimized wavelength. In order to evaluate the bandwidth characteristics, we employ two parameters: the mean cross-talk and the maximum cross-talk, which are respectively defined as,

$$\begin{cases} W_1 = \underset{\alpha,\beta\&\alpha\neq\beta}{\text{mean}}\left\{P_{\alpha,\beta}\right\} \\ W_2 = \underset{\alpha,\beta\&\alpha\neq\beta}{\max}\left\{P_{\alpha,\beta}\right\} \\ P_{\alpha,\beta} = \dfrac{p_{out}^{\alpha,\beta}}{\sum_\beta p_{out}^{\alpha,\beta}} \end{cases}, \tag{S15}$$

where $p_{out}^{\alpha,\beta}$ represents the energy of the β-*th* channel at the case of α-*th* input mode, and $P_{\alpha,\beta}$ denotes the normalized energy of the β-*th* channel for the α-*th* input mode. The results for the 5-layer system are shown in Figure S5.

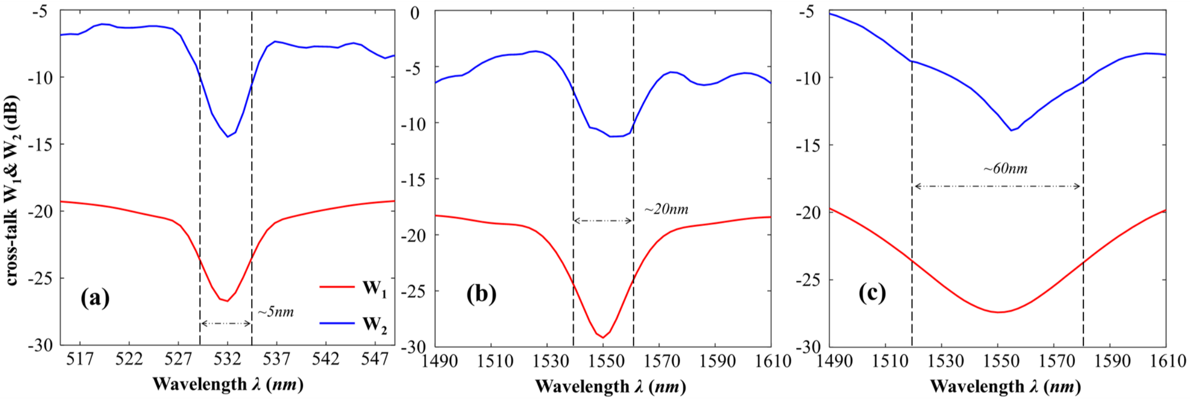

Figure S5. The broadband characteristics of the system.

As depicted in Figure S5 (a), the bandwidth of the mode sorter is approximately 5 nm. This narrower bandwidth in our study, compared to existing works[5], can be ascribed to the utilization of a smaller wavelength (532 nm). Indeed, the variation in wavelength $\delta\lambda$ profoundly influences the phase, namely, $\varphi\frac{\lambda_0}{\lambda^2}\delta\lambda$, which is approximately inversely proportional to the central wavelength. With an increased wavelength such as the communication band around 1550 nm, the bandwidth will extend from 5 nm to 20 nm, as illustrated in Figure S5 (b).

In fact, there is a straightforward method to amplify the broadband. Borrowing from the design principle of an achromat lens, we introduce a Gaussian random variable $\Delta\lambda$ for the wavelength during training, while maintaining other parameters consistent with those in Figure S5 (b). This implies that the wavelength experiences variation during the training process, culminating in broadband enhancement. As demonstrated in Figure S5 (c), the broadband can be further broadened from 10 nm to 30 nm using this simplistic approach. Moreover, fine-tuning the loss function to accommodate the broadband could potentially yield significant enhancements.

[1] J. W. Goodman, *Introduction to Fourier Optics*, 3rd ed. (Roberts and Company Publishers, Englewood, 2005).
[2] T. Zhou, L. Fang, T. Yan, J. Wu, Y. Li, J. Fan, H. Wu, X. Lin, and Q. Dai, Photonics Research **8**, 940 (2020).
[3] E. Bolduc, N. Bent, E. Santamato, E. Karimi, and R. W. Boyd, Optics Letters **38**, 3546 (2013).
[4] T. W. Clark, R. F. Offer, S. Franke-Arnold, A. S. Arnold, and N. Radwell, Optics Express **24**, 6249 (2016).
[5] N. K. Fontaine, R. Ryf, H. Chen, D. T. Neilson, K. Kim, and J. Carpenter, Nature Communications **10**, 1865 (2019).
